# A242 SCLEROSING MESENTERITIS OF THE PORTA HEPATIS PRESENTING AS CHOLANGIOCARCINOMA WITH RECURRENT CHYLOUS ASCITES AND GASTRIC OUTLET OBSTRUCTION

**DOI:** 10.1093/jcag/gwae059.242

**Published:** 2025-02-10

**Authors:** T T Hoang, A Karavelic, A Sunil, T Murray, J Telford, E Clement, A Gador

**Affiliations:** The University of British Columbia Faculty of Medicine, Vancouver, BC, Canada; The University of British Columbia Faculty of Medicine, Vancouver, BC, Canada; The University of British Columbia Faculty of Medicine, Vancouver, BC, Canada; The University of British Columbia Faculty of Medicine, Vancouver, BC, Canada; The University of British Columbia Faculty of Medicine, Vancouver, BC, Canada; The University of British Columbia Faculty of Medicine, Vancouver, BC, Canada; The University of British Columbia Faculty of Medicine, Vancouver, BC, Canada

## Abstract

**Background:**

Sclerosing mesenteritis (SM) is characterized by chronic fibroinflammatory infiltration of the abdominal mesentery. While commonly affecting the small bowel, we present the first reported case of SM mimicking hilar cholangiocarcinoma by involving the porta hepatis, resulting in chylous ascites, gastric outlet obstruction, and duodenal perforation.

**Aims:**

To broaden recognition of this rare disease and expand literature on treatment options

**Methods:**

Case report and literature review

**Results:**

A 59-year-old man with previous alcoholic pancreatitis presented with one month of progressive abdominal pain and distension. He was found to have large volume chylous ascites and a hilar mass on computed tomography scan. Magnetic resonance cholangiopancreatography confirmed a soft tissue density at the porta hepatis with intra- and extrahepatic duct dilation. Endoscopic ultrasound (EUS) revealed a soft tissue mass and biopsies showed a chronic lymphohistiocytic infiltrate but no malignancy. Positron emission tomography, liver biopsy, and bidirectional endoscopy were unrevealing. Serial large-volume paracenteses were performed for comfort, though portal hypertension was ruled out via transjugular measurements. One month into hospitalization, he developed gastric outlet obstruction from extrinsic mass and required nasojejunal feeding. This was complicated by a contained duodenal perforation managed non-operatively. Palliative dexamethasone was started for presumed cholangiocarcinoma which entirely resolved his symptoms. He was discharged after three months but represented off steroids with recurrent ascites and gastric outlet obstruction. Diagnostic laparoscopy was negative. EUS-guided biopsy again showed the same chronic inflammatory infiltrate. He was diagnosed with SM and started on oral prednisone which resolved his symptoms. He remains in remission as an outpatient on colchicine, tamoxifen, and low-dose prednisone.

**Conclusions:**

SM is a rare disease characterized by chronic mesenteric inflammation, fibrosis, and fat necrosis. While 10% of cases are incidental findings, complications occur in 24% of patients and include recurrent chylous ascites, small bowel obstruction, and intestinal perforation. Diagnosis is challenging given non-specific findings but can be made on histology and radiology. This benign disease is often mistaken for malignancy, resulting in unnecessary diagnostic tests. To our knowledge, this is only the second reported case of hilar SM, and the first to present with the aforementioned complications. Treatment includes prednisone and tamoxifen, with colchicine, azathioprine, or thalidomide used as steroid-sparing agents. Increased recognition of this entity will help avoid unnecessary diagnostic interventions and inform options for medical therapy.

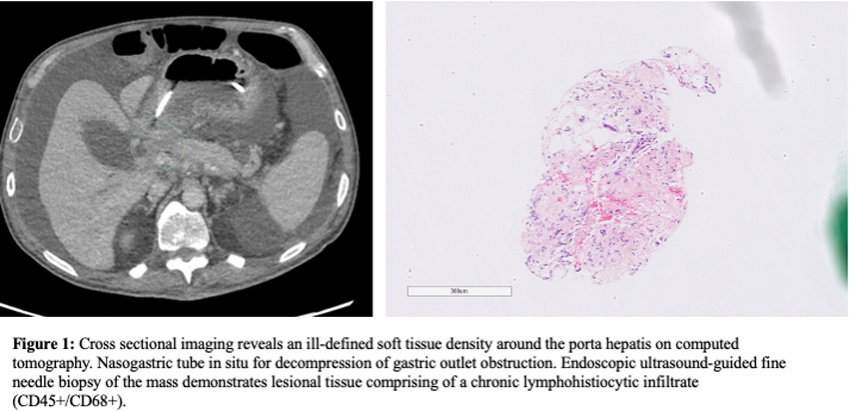

**Funding Agencies:**

None

